# Cannabinoid CB_1_ Receptor Activation Mitigates *N*‑Methyl‑d‑aspartate Receptor-Mediated Neurotoxicity

**DOI:** 10.1021/acsptsci.5c00230

**Published:** 2025-08-25

**Authors:** Gemma Navarro, Iu Raïch, Joan Biel Rebassa, Jaume Lillo, Catalina Pérez-Olives, Toni Capó, Erik Cubeles, Carlos A. Saura, Arnau Cordomí, Eddy Sotelo, Irene Reyes-Resina, Rafael Franco

**Affiliations:** † Centro de Investigación Biomédica en Red Enfermedades Neurodegenerativas (CiberNed), National Institute of Health Carlos III, 28031 Madrid, Spain; ‡ Institut de Neurociències UB, Campus Mundet, 08035 Barcelona, Spain; § Department of Biochemistry and Physiology, Faculty of Pharmacy and Food Science, 16724 University of Barcelona, 08028 Barcelona, Spain; ∥ Departament de Biochemistry and Molecular Biomedicine, 16724 University of Barcelona, 08028 Barcelona, Spain; ⊥ Institut de Neurociències, Department de Bioquímica i Biologia Molecular, Facultat de Medicina, Universitat Autònoma de Barcelona, Bellaterra, 08193 Barcelona, Spain; # Departament de Bioquímica i de Biologia Molecular, Universitat Autònoma de Barcelona, Bellaterra, 08193 Barcelona, Spain; 7 Centro Singular de Investigación en Química Biolóxica e Materiais Moleculares (CiQUS), 15705 Santiago de Compostela, Spain; 8 Institut de Química Teòrica i Computacional (IQTCUB), School of Chemistry, University of Barcelona, 08028 Barcelona, Spain

**Keywords:** NMDA receptor, receptor−receptor interaction, excitotoxicity, functional selectivity, neuroprotection, Alzheimer’s disease, signalosome

## Abstract

Alzheimer’s disease (AD) is characterized by synaptic dysfunction and excitotoxicity, yet effective therapeutic strategies remain limited. This study explores the functional and physical interplay between cannabinoid CB_1_ receptors (CB_1_Rs) and *N*-methyl-d-aspartate receptors (NMDARs), which are implicated in AD pathology. Using bioluminescence resonance energy transfer and imaging assays in HEK-293T cells, we demonstrate a direct interaction between CB_1_R and the N1 subunit of NMDAR, supporting the formation of receptor complexes. Functional assays further reveal a bidirectional negative crosstalk: NMDA attenuates CB_1_R-mediated cAMP inhibition, while CB_1_R activation reduces NMDA-induced calcium influx and mitogen-activated protein kinase signaling pathway activation. This negative crosstalk suggests the existence of receptor–receptor interactions with functional consequences. Complexes of CB_1_Rs and N_1_ subunits of NMDARs are present in both neurons and microglia, and their expression is upregulated in response to Aβ_1–42_ and in cells derived from the APP_Sw/Ind_ AD model mice. However, upregulation did not always correlate with stronger CB_1_R–NMDAR cross-modulation, suggesting that cell-specific signalosome composition shapes the signaling outcome. Functionally, CB_1_R activation confers neuroprotection: It rescues neurite loss induced by NMDA and Aβ_1–42_, highlighting the therapeutic potential of modulating CB_1_R–NMDAR interactions. These findings support a model in which CB_1_R–NMDAR interactions, through dynamic functional cross-modulation, finely tune excitotoxic and inflammatory signaling pathways. This mechanism offers therapeutic prospects for addressing cannabinoid-glutamatergic interactions.

## Graphical abstract

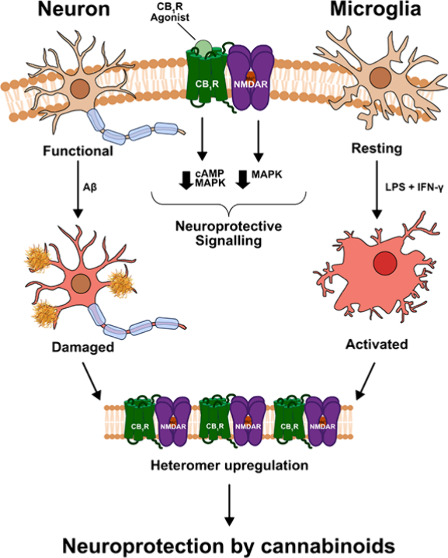

Alzheimer’s disease (AD) was first described by Alois Alzheimer in 1906 and nowadays represents 60 to 80% of the 55 million cases of dementia around the world.^1^ There are two forms of AD; the sporadic form is characterized by a not fully understood etiopathogenesis and by the influence of different factors such as lifestyle, environment, and other genetic and epigenetic alterations. It accounts for about 95% of AD cases. The familial AD is caused by mutations in the amyloid precursor protein (APP) and in the presenilin-1 and -2 genes and represents around 5% of cases.^2^ The fact that AD still does neither have a cure not interventions to delay disease progression leads to the necessity of discovering new therapeutic targets.

AD begins with the loss of ability to retain new memories and progresses with different cognitive and behavioral changes, which worsen over time. The pathophysiology of AD is characterized by aggregates of amyloid precursor protein (APP)-derived ß-amyloid (Aβ) peptides and hyperphosphorylated tau protein.^3,4^ Overall, synaptic dysfunction and neuronal death generally result in progressive neurodegeneration.^5^ Neurodegeneration is accompanied by alterations in synaptic plasticity that, at least, is partially mediated by altered glutamatergic neurotransmission. In fact, *N*-methyl-d-aspartate (NMDA) and α-amino-3-hydroxy-5-methyl-4-isoxazole propionic acid (AMPA) glutamate receptors are directly involved in synaptic plasticity.^6^

The NMDA glutamate receptor (NMDAR) is an ion channel that requires the binding of two coagonists for activation: glutamate and either glycine or d-serine. The receptor is typically formed by a tetramer consisting of two N1 and two N2 subunits. Glutamate binds to the N2 subunits, while glycine or d-serine bind to the N1 subunits. In addition to ligand binding, channel opening also requires relief of a voltage-dependent magnesium block, which occurs upon membrane depolarization.^7^ Glutamate is the main excitatory neurotransmitter in the human brain. However, sustained elevations in extracellular glutamate levels can lead to excitotoxicity and neurodegeneration. Synaptic NMDARs, primarily containing N2A subunits, are generally associated with cell survival and neuroprotective signaling, whereas extrasynaptic NMDARs, often containing N2B subunits, are linked to neuronal vulnerability and excitotoxic pathways.^8^

NMDAR function may be regulated by neurotransmitters or neuromodulators acting via G protein-coupled receptors (GPCRS).^9–11^ The activation of adenosine A_2A_ receptors modulates NMDAR functionality likely by a direct interaction of NMDAR and adenosine A_2A_ receptors.^10^ NMDARs may also interact with dopamine D_1_ and histamine H_3_ receptors, forming NMDAR-D_1_R-H_3_R functional complexes.^9^ The regulation of NMDARs by interaction with cannabinoid receptors is relevant in the AD context. NMDAR may interact with the cannabinoid CB_2_ receptor (CB_2_R), and evidence of the interaction was found both in primary cultures of neurons and microglia and in brain sections.^12^ The print of NMDAR–CB_2_R interaction consists of an impairment of NMDAR function by CB_2_R activation. This feature was detected in microglial cells activated with LPS plus IFN-γ, and in primary microglia from the brain of APP_Sw/Ind_ mice, a transgenic model of AD.^13^

Cannabinoids bind to two different receptors: CB_1_ and CB_2_. In the central nervous system, CB_2_Rs are mainly expressed in glia, whereas CB_1_ receptors (CB_1_Rs) are expressed in both neurons and glia. In humans, the cannabinoid CB_1_R is primarily expressed in the cortex, hippocampus, cerebellum, basal ganglia, and brainstem.^14^ Although its pathophysiological role in AD remains unclear, the genetic deletion of the CB_1_Rs associates with a loss of neurons and a cognitive deficit in the hippocampus of adult KO mice.^15^ Interestingly, acute activation of CB_1_R, particularly at a young age, impairs short-term memory in a dose-dependent manner.^16^ Multiple studies have demonstrated that CB_1_Rs can counterbalance the effects of NMDA receptors (NMDARs), thereby contributing to the neuroprotective role of cannabinoids against excessive NMDAR activation.^17–19^ This interaction plays a role in modulating nociceptive signaling under glutamatergic stimulation, as disruption of CB_1_R function has been shown to result in heightened NMDAR-mediated pain responses, including allodynia and hyperalgesia.^20^ Importantly, in neuropathic conditions characterized by NMDAR overactivity, which typically reduces the efficacy of potent analgesics like opioids, cannabinoids may still retain partial analgesic activity.^21^

While CB_1_Rs are well-known for their presynaptic localization, they are also present postsynaptically,^22^ where they overlap with the expression of NMDARs. In fact, N1 subunits of NMDARs colocalize with CB_1_Rs on neuronal somata and dendrites in the rat hippocampus.^23^ In addition, Sánchez-Blázquez et al. reported that CB_1_R coprecipitates with the N1 subunit, but not—or only minimally—with the N2 subunit. Notably, this interaction was reduced following NMDAR overactivation.^24^ Here, we demonstrate a molecular interaction between the CB_1_R and subunits of the NMDAR in transfected HEK-293T cells, primary neurons and primary microglia. Functional signaling data, such as ERK1/2 phosphorylation and calcium responses, obtained in these cells as well as in primary microglia and neurons, show a crosstalk between CB_1_ and NMDA receptors. Furthermore, the characteristics of the inter-receptor modulation vary from cell to cell and are context dependent.

In this study, we aimed to investigate the molecular and functional interaction between CB_1_ receptors and NMDARs in neurons and microglia, with a particular focus on their modulation by Aβ_1‑42_ and in the context of AD. By combining biochemical, imaging, and functional approaches, we sought to determine how CB_1_R–NMDAR complexes contribute to the regulation of excitotoxic and inflammatory signaling pathways, and to evaluate their potential as neuroprotective targets.

## Materials and Methods

### Reagents

LPS, interferon-γ (IFN-γ), and Aß_1–42_ peptide were purchased from Sigma-Aldrich (St Louis, Missouri), and ACEA, rimonabant, NMDA, and MK-801 were purchased from Tocris Bioscience (Bristol, UK).

### Transgenic Mouse Model of Alzheimer’s Disease (AD)

APP_Sw/Ind_ transgenic mice (line J9; C57BL/6 background) expressing human APP695 harboring the familial Alzheimer′s disease-linked Swedish (K670N/M671L) and Indiana (V717F) mutations under the PDGFβ promoter were obtained by crossing heterozygous APP_Sw/Ind_ with nontransgenic animals.^25^ Mice at 2 days of age were genotyped individually by conventional PCR.^26,27^ Experimental procedures were conducted according to the Animal and Human Ethical Committee of the Universitat Autònoma de Barcelona (protocol CEEAH 1783, Generalitat Catalunya 6381) following the European Union guidelines. Experiments with primary cultures (see below) were performed blindly, without knowing the genotype, which was disclosed for data analysis.

### Aß_1–42_ Oligomer Production

Human Aß-oligomers (Sigma-Aldrich, St Louis, Missouri) were prepared according to previously established protocol.^28^ Briefly, the lyophilized Aß_1–42_ was disaggregated in 1,1,1,3,3,3-hexafluoro-2-propanol (HFIP, Sigma-Aldrich, St Louis, Missouri, USA, Cat. No. B2517) to 0.5 mg/mL. Then, the HFIP was evaporated at room temperature and stored at −80 °C. 24 h before the experiment, the peptide was dissolved in DMSO (Sigma-Aldrich, 1/1000) and then sonicated. Finally, the peptide was diluted to 50 μM in F12 medium (Gibco) and kept at 4 °C for 24 h to allow oligomerization. For each experiment, a fresh oligomer preparation was used.

### Expression Vectors

The cDNA for the human version of cannabinoid CB_1_R without its stop codon was obtained by PCR and subcloned to a SNAP-containing vector (PSNAP; Cisbio Bioassays) and YFP-containing vector (pEYFP-N1, PerkinElmer, Wellesley, Massachusetts) using sense and antisense primers harboring unique restriction sites for *Hin*dIII and *Bam*HI, generating the SNAP- and YFP-tagged CB_1_R (SNAP-CB_1_R and CB_1_R-YFP). The cDNA for the human version of the N1 subunit of the NMDA receptor without its stop codon was obtained by PCR and subcloned to the Rluc-containing vector (p*Rluc*-N1; PerkinElmer, Wellesley, Massachusetts) using sense and antisense primers harboring unique restriction sites for *Hin*dIII and *Bam*HI, generating N1-Rluc.

### Cell Culture and Transfection

HEK-293T cells (lot #70022180) were acquired from the American Type Culture Collection (ATCC). Cells were amplified and grown until passage 18 in Dulbecco’s modified Eagle’s medium (DMEM) supplemented with 2 mM l-glutamine, 100 U/mL penicillin/streptomycin, and 5% (v/v) heat-inactivated fetal bovine serum (FBS) (Invitrogen, Paisley, Scotland, UK). Cells were maintained in a humid atmosphere of 5% CO_2_ at 37 °C. Cells were transiently transfected with the PEI (polyethylenimine, Sigma, St. Louis, Missouri, USA) method, as previously described (Navarro et al., 2015). To prepare mouse primary microglia, the brain was removed from P2–P3 C57BL/6 mouse pups. Microglial cells were isolated as described in ref 29 and plated at confluence of 40,000 cells/0.32 cm^2^ and grown in DMEM supplemented with 2 mM l-glutamine, 100 U/mL penicillin/streptomycin, and 5% (v/v) heat-inactivated fetal bovine serum (FBS) (Invitrogen, Paisley, Scotland, UK) for 12 days. For preparing primary neurons of mice, cortex and hippocampus were removed from the brain of from E18–E19 fetuses. Neurons were isolated as described in ref 30 and plated at a confluence of 40,000 cells/0.32 cm^2^. Neuronal cells were grown in neurobasal medium supplemented with 2 mM l-glutamine, 100 U/mL penicillin/streptomycin, and 2% (v/v) B27 supplement (Gibco) in a 96-well plate for 12 days.

### HTRF Assay

HTRF assays were performed in transfected HEK-293T cells expressing SNAP-tagged CB_1_R in the presence or in the absence of NMDAR. Tag-lite-based binding assays were performed 24 h after transfection. For cell labeling, culture medium was removed followed by the addition of 100 nM SNAP-Lumi4-Tb, previously diluted in 3 mL of TLB 1× for 1 h at 37 °C under a 5% CO_2_ atmosphere in a cell incubator. Cells were then washed four times with 2 mL of TLB 1× to remove the excess of SNAPLumi4-Tb, detached with enzyme-free cell dissociation buffer, centrifuged for 5 min at 1500 rpm and collected in 1 mL of TLB 1×. Densities in the 2500–3000 cells/well range were used to carry out binding assays in white opaque 384-well plates.

The fluorophore-conjugated CB_1_R ligand (labeled CELT-335) and unconjugated CB_1_R agonist, ACEA, were diluted in TLB 1×. HEK-293T cells transiently expressing Tb-labeled SNAP-CB_1_R with or without NMDAR were incubated with a 20 nM fluorophore-conjugated CB1R ligand, in the presence of increasing concentrations (0–10 mM range) of ACEA. Plates contained 10 mL of labeled cells, and 5 mL of TLB 1× or 5 mL of ACEA was added prior to the addition of 5 mL of the fluorescent ligand. Plates were then incubated for at least 2 h at room temperature before signal detection. A detailed description of the HTRF assay is found in ref 31. Signal was detected using a PHERAstar FS (BMG Lab Technologies, Offenburg, Germany) microplate reader equipped with a FRET optic module allowing donor excitation at 337 nm and signal collection at both 665 and 620 nm. A frequency of 10 flashes/well was selected for the xenon flash lamp excitation. The signal was collected at both 665 and 620 nm using the following time-resolved settings: delay, 150 ms; integration time, 500 ms. HTRF ratios were obtained by dividing the acceptor (665 nm) by the donor (620 nm) signals and multiplying by 10,000. The 10,000-multiplying factor is used solely for the purpose of easier data handling.

### Bioluminescence Resonance Energy Transfer (BRET) Assay

HEK-293T cells were transiently cotransfected with a constant amount of cDNA encoding for N1-Rluc and N2B in pcDNA3.1 or dopaminergic D_1_R-Rluc as negative control and with increasing amounts of cDNA corresponding to CB_1_R-YFP. 48 h after transfection, cells were adjusted to 20 μg of protein using a Bradford assay kit (Bio-Rad, Munich, Germany), using bovine serum albumin for standardization. To quantify protein-YFP expression, fluorescence was read in a Mithras LB 940 equipped with a high-energy xenon flash lamp, using a 30 nm bandwidth excitation filter at 485 nm reading. For BRET measurements, readings were collected 1 min after the addition of 5 μM coelenterazine H (Molecular Probes, Eugene, Oregon) using a Mithras LB 940, which allows the integration of the signals detected in the short-wavelength filter at 485 nm and the long-wavelength filter at 530 nm. To quantify protein-Rluc expression, luminescence readings were performed 10 min after 5 μM coelenterazine H addition using a Mithras LB 940.

### Immunocytochemistry

HEK-293T cells were transfected with N1-Rluc, N2B, and CB_1_R-YFP, fixed in 4% paraformaldehyde for 15 min, and washed twice with PBS containing 20 mM glycine before permeabilization with PBS-glycine containing 0.2% Triton X-100 (5 min incubation). HEK-293T cells were treated for 1 h with PBS containing 1% bovine serum albumin, labeled with the primary mouse anti-Rluc antibody, and subsequently treated with a Cy3-conjugated antimouse (1/200; Jackson ImmunoResearch (red)) secondary antibody for 1 h. The CB_1_R-YFP fusion protein was detected by YFP’s own fluorescence. Colocalization is observed in yellow. Nuclei were labeled with Hoechst (blue). Samples were washed several times and mounted with 30% MOWIOL (Calbiochem). Samples were observed under a Zeiss 880 confocal microscope (Carl Zeiss, Oberkochen, Germany). Scale bar: 10 μm.

### cAMP Level Determination

cAMP experiments were performed as previously described.^32,33^ HEK-293T cells were transfected with the cDNAs for N1 (0.5 μg/well), N2B (0.5 μg/well), and CB_1_R (0.3 μg/well). Two hours before initiating the experiment, HEK-293T, neuronal, or microglial cell culture medium was replaced by serum-starved DMEM medium. Then, cells were detached, resuspended in growing medium containing 50 mM zardaverine, and placed in 384-well microplates (2500 cells/well). Cells were pretreated (15 min) with selective antagonists (SR141617 for CB_1_R or MK-801 for NMDAR) followed by agonist stimulation (ACEA for CB_1_R and/or NMDA for NMDAR) or vehicle for an additional period of 15 min before adding 0.5 mM forskolin (FK) or vehicle. Readings were performed after 60 min of incubation at 25 °C. HTRF energy transfer measures were performed using the Lance Ultra cAMP kit (PerkinElmer, Waltham, Massachusetts, United States). Fluorescence at 665 nm was analyzed in a PHERAstar Flagship microplate reader equipped with an HTRF optical module (BMG Lab Technologies, Offenburg, Germany).

### ERK Phosphorylation Assays

To determine the phosphorylation of extracellular signal-regulated kinase 1/2 (ERK1/2), 50,000 HEK-293T cells/well, neurons, or microglia were plated in transparent Deltalab 96-well microplates and kept at the incubator for 24 h. HEK-293T cells were transfected with the cDNAs for N1 (0.5 μg/well), N2B (0.5 μg/well), and CB_1_R (0.3 μg/well). 2 to 4 h before the experiment, the medium was substituted by serum-starved DMEM. Then, cells were pretreated at 25 °C for 10 min with selective antagonists (SR141617 for CB_1_R or MK-801 for NMDAR) followed by agonist stimulation (ACEA for CB_1_R and/or NMDA for NMDAR) or vehicle. Cells were then washed twice with cold PBS before the addition of lysis buffer (20 min treatment). Ten microliters of each supernatant was transferred to white ProxiPlate 384-well microplates, and ERK1/2 phosphorylation was quantified using the AlphaScreen SureFire Kit (PerkinElmer, Waltham, Massachusetts, USA), following the manufacturer’s instructions. Signal detection was performed using an EnSpire Multimode Plate Reader (PerkinElmer).

To assess ERK1/2 phosphorylation in hippocampal and cortical slices, tissues were maintained in HBSS supplemented with glucose for 2 h at 37 °C and then stimulated for 15 min with selective agonists. Samples were sonicated on ice (2 × 10 s pulses) in lysis buffer, and the total protein concentration was adjusted to 1 μg/μL using SDS and lysis buffer. Equal amounts of protein (20 μg) were separated by electrophoresis on 10% SDS–polyacrylamide gels and transferred to PVDF membranes (Immobilon-FL, Merck, St. Louis, Missouri, USA) using the Trans-Blot Turbo system (Bio-Rad) for 30 min. Membranes were blocked for 2 h at room temperature with constant shaking using Odyssey Blocking Buffer (LI-COR Biosciences, Lincoln, Nebraska, USA) and incubated overnight at 4 °C with a primary antibody mix containing mouse antiphospho-ERK1/2 (1:2500; Merck, ref. M8159) and rabbit antitotal ERK1/2 (1:40,000; Merck, ref. M5670). After washing with 0.05% PBS-Tween, membranes were incubated for 2 h at room temperature (protected from light) with IRDye 800CW antimouse (1:10,000; Merck, ref. 926-32210) and IRDye 680RD antirabbit (1:10,000; Merck, ref. 926-68071) secondary antibodies. Membranes were washed again with PBS-Tween, and bands were visualized using an Odyssey Infrared Imaging System (LI-COR Biosciences). Band intensities were quantified using Fiji software. Phospho-ERK1/2 levels were normalized to total ERK1/2, and results are expressed as percentage relative to basal (vehicle-treated) conditions.

### Dynamic Mass Redistribution (DMR) Assays

Cell mass redistribution induced upon receptor activation was detected by illuminating the underside of a biosensor with polychromatic light and measuring the changes in the wavelength of the reflected monochromatic light. The magnitude of this wavelength shift (in picometers) is directly proportional to the amount of DMR. HEK-293T cells expressing the cDNAs for N1, N2B and CB_1_R were seeded in 384-well sensor microplates to obtain 70–80% confluent monolayers constituted by approximately 10,000 cells per well. Previous to the assay, cells were washed twice with assay buffer (HBSS with 20 mM HEPES, pH 7.15) and incubated for 2 h with assay buffer containing 0.1% DMSO (24 °C, 30 mL/well). Hereafter, the sensor plate was scanned and a baseline optical signature was recorded for 10 min before adding 10 mL of the selective antagonists (SR141617 for CB_1_R or MK-801 for NMDAR) for 30 min followed by the addition of 10 mL of specific agonists (ACEA for CB_1_R and/or NMDA for NMDAR) or vehicle; all test compounds were dissolved in assay buffer. The cell signaling signature was determined using an EnSpire Multimode Plate Reader (PerkinElmer, Waltham, Massachusetts, United States) by a label-free technology. Then, DMR responses were monitored for at least 5,000 s. Results were analyzed using an EnSpire Workstation Software v 4.10.

### ß-Arrestin-2 Recruitment

ß-Arrestin-2 recruitment was determined as previously described.^33^ Briefly, BRET experiments were performed in HEK-293T cells 48 h after transfection with the cDNAs corresponding to the CB_1_R-YFP (1 μg cDNA), N1 (05 μg cDNA), N2B (0,5 μg cDNA), and ß-arrestin-2-Rluc (0,5 μg cDNA). Cells (20 mg protein) were distributed in 96-well microplates (Corning 3600, white plates with white bottom) and were incubated with 10 mL of selective antagonists (SR141617 for CB_1_R or MK-801 for NMDAR) for 10 min followed by the addition of 10 mL of specific agonists (ACEA for CB_1_R and/or NMDA for NMDAR) or vehicle 1 min before adding coelenterazine H (0.5 μM). BRET between ß-arrestin-2-Rluc and CB_1_R-YFP was immediately determined and quantified. The readings were collected using a Mithras LB 940 (Berthold Technologies, Bad Wildbad, Germany) that allows the integration of the signals detected in the short-wavelength filter at 485 nm and the long-wavelength filter at 530 nm. To quantify CB_1_R-Rluc expression, luminescence readings were also performed 10 min after adding 5 μM coelenterazine H.

### Cytosolic Calcium Determination

HEK-293T cells were cotransfected with the cDNA for CB_1_R (0.3 μg/well), N1 (0.5 μg/well), N2B (0.5 μg/well), and 0.5 μg/well of the GCaMP6 calcium sensor^34^ using PEI protocol (see Cell Culture and Transfection). 48 h after transfection, cells (150,000 HEK-293T cells/well in 96-well black, clear bottom microtiter plates) were incubated with Mg^2+^-free Locke’s buffer pH 7.4 (154 mM NaCl, 5.6 mM KCl, 3.6 mM NaHCO_3_, 2.3 mM CaCl_2_, 5.6 mM glucose, and 5 mM HEPES) supplemented with 10 μM glycine and receptor antagonists (SR141617 for CB_1_R or MK-801 for NMDAR) for 10 min followed by agonists stimulation (ACEA for CB_1_R and/or NMDA for NMDAR) just a few seconds before readings. The fluorescence emission intensity of GCaMP6 was recorded at 515 nm upon excitation at 488 nm on the EnSpire Multimode Plate Reader for 450 s every 15 s and 100 flashes per well.

### Proximity Ligation Assays (PLA)

The interaction between NMDA and CB_1_R was detected using the Duolink II In Situ PLA Detection Kit (OLink; Bioscience, Uppsala, Sweden) following the instructions of the supplier. Primary neurons and microglial cells were grown on glass coverslips and were fixed in 4% paraformaldehyde for 15 min, washed with PBS containing 20 mM glycine to quench the aldehyde groups, permeabilized with the same buffer containing 0.05% Triton X-100 for 5 min, and successively washed with PBS. After 1 h of incubation at 37 °C with the blocking solution in a preheated humidity chamber, primary cells were incubated overnight in the antibody diluent medium with a mixture of equal amounts of rat monoclonal anti-N1 antibody (1:200, Millipore) and a polyclonal rabbit anti-CB_1_R antibody (1:100, Abcam). Cells were processed using the PLA probes detecting primary antibodies (Duolink II PLA probe plus and Duolink II PLA probe minus) diluted in the antibody diluent to a concentration of 1:5. Ligation and amplification were done as indicated by the supplier and cells were mounted using the mounting medium with Hoechst (1/200; Sigma). Samples were observed under a Zeiss 880 confocal microscope (Carl Zeiss, Oberkochen, Germany) equipped with an apochromatic 63× oil-immersion objective (N.A. 1.4) and 405 nm and 561 nm laser lines. For each field of view, a stack of two channels (one per staining) and four to eight Z stacks with a step size of 1 μm were acquired. A quantification of cells containing one or more red spots versus total cells (blue nucleus) and, in cells containing spots, the ratio *r* (number of red spots/cell) were determined. One-way ANOVA followed by Bonferroni’s *post hoc* multiple comparison test was used to compare the values (% of positive cells or *r* spots/cell) obtained for each pair of receptors.

### Neurite Patterning Determination

Cortical and hippocampal primary neurons seeded for 11 days were treated with Aβ (500 nM) for 48 h and subsequently stimulated with NMDA (15 μM), ACEA (100 nM), or vehicle for 24 h. Then, cells were fixed in 4% paraformaldehyde for 15 min and then washed twice with PBS containing 20 mM glycine followed by permeabilization with the same buffer containing 0.2% Triton X-100 (15 min incubation). Samples were treated for 1 h with blocking solution (PBS containing 1% bovine serum albumin) and labeled with polyclonal rabbit anti-Nectin 3 antibody (Abcam, 1/1000). Neurons were detected with 3 anti-F-actin antibody fused to an Alexa 488 fluorophore (Thermo Fisher, 1/400). Then, samples were incubated at RT for 2 h with a Cy3-conjugated antirabbit secondary antibody (1/200, 711-166-152, Jackson ImmunoResearch). Finally, cells were washed several times with PBS and mounted with 30% MOWIOL (Calbiochem, San Diego, California, USA). Nuclei were stained with Hoechst (1/100). Samples were observed under a Zeiss 880 confocal microscope (Leica Microsystems, Wetzlar, Germany). Quantification of neurite formation was performed over segments of 15 μm. Each red dot represents a neurite formation.

### Data Handling and Statistical Analysis

Data from homogeneous binding assays were analyzed using Prism 6 (GraphPad Software, Inc., San Diego, California, United States). *K*_*i*_ values were determined according to the Cheng and Prusoff equation (Cheng, 2001). Signal-to- background (S/B ratio) calculations were performed by dividing the mean of the maximum value (μ_max_) by that of the minimum value (μ_min_) obtained from the sigmoid fits. The data are shown as the mean ± SEM. Statistical analysis was performed with Prism 8.0 software. Significance was analyzed by one-way ANOVA, followed by Bonferroni’s multiple comparison post hoc test. Significant differences were considered when *p* < 0.05.

## Results

### Cannabinoid CB_1_ Receptor Shows Lower Affinity for Its Agonist when Forming Complexes with NMDAR

Colocalization (in yellow) between CB_1_R and the N1 subunit of the NMDA receptor was detected in HEK-293T cells expressing CB_1_R-YFP, N1-Rluc, and N2B (Figure 1A). CB_1_R-YFP was detected by the YFP’s own fluorescence (shown in green), and the Cy3-conjugated antimouse secondary antibody was used to label the anti-Rluc primary antibody (shown in red). Next, Bioluminescence Resonance Energy Transfer (BRET) assays were performed in HEK-293T cells expressing a constant amount of N1-Rluc and N2B and increasing amounts of CB_1_R-YFP. A saturation BRET curve that demonstrates the physical interaction between the two proteins was obtained (Figure 1B). A similar experiment was performed in cells expressing a constant amount of dopamine D_1_ receptors fused to Rluc and increasing amounts of CB_1_R-YFP. The linear relationship observed (Figure 1B) indicates no interaction between the two proteins, validating the condition as a negative control.

**Figure 1:**
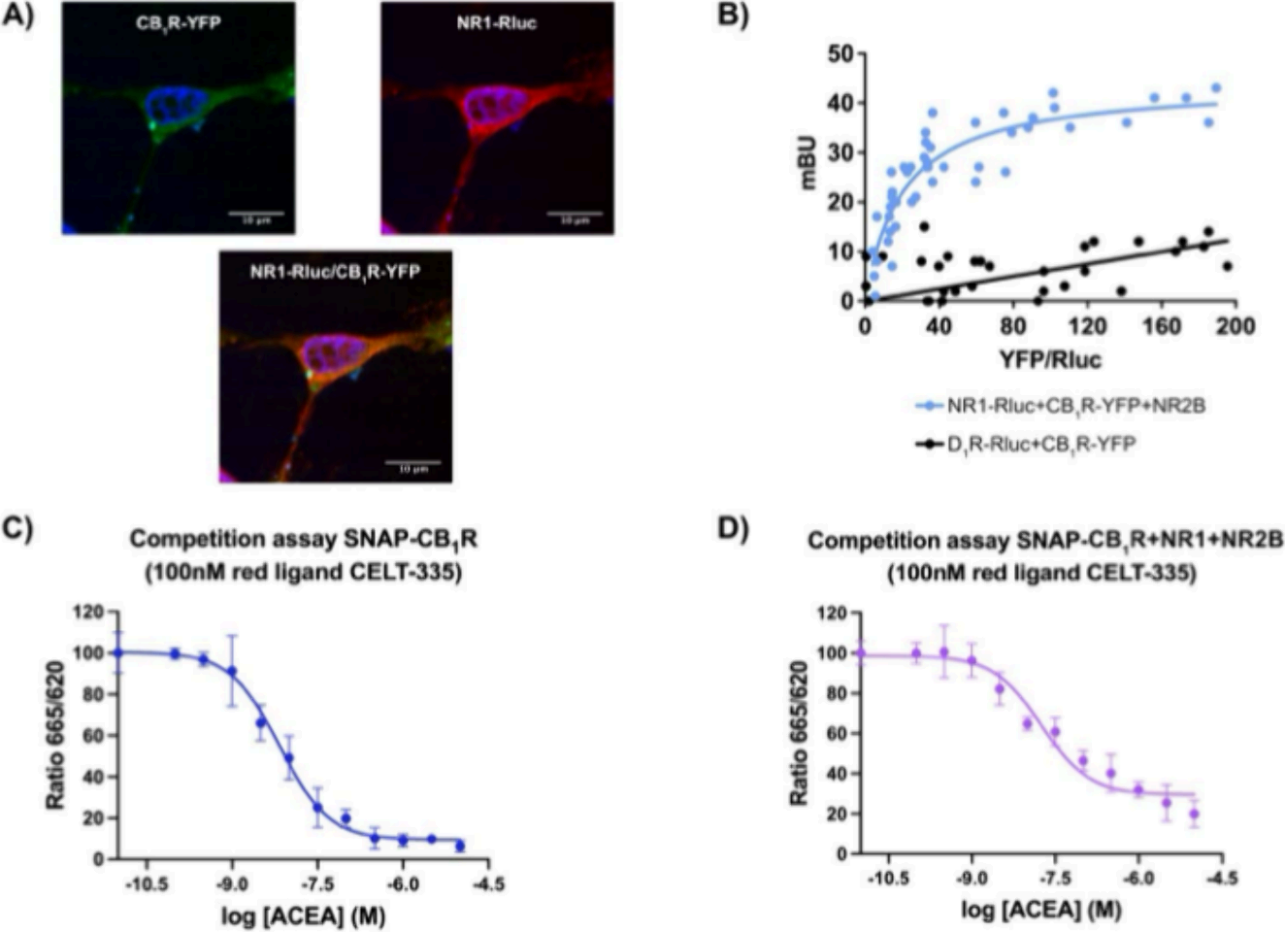
Evidence of CB_1_R and NMDAR interaction in transfected HEK-293T cells. (A) Immunocytochemistry assay was performed in HEK-293T cells expressing CB_1_R-YFP, which was detected by YFP’s own green fluorescence (green), the N2B subunit, and N1-Rluc, which was detected by a mouse anti-Rluc antibody (red). Nuclei were stained with Hoechst (blue). Colocalization is shown in yellow. Scale bar: 10 μm. (B) BRET assays were performed in HEK-293T cells expressing constant amounts of N1-Rluc and N2B (or D_1_R-Rluc) and increasing amounts of CB_1_R-YFP. Values are the mean ± SEM of sx different experiments performed in triplicates. (C, D) HTRF-based competitive binding assays were performed in HEK-293T cells expressing SNAP-CB_1_R in the absence (C) or presence of N1 and N2B (D). Competition binding curves for ACEA were obtained using 20 nM CELT-335 fluorescent ligand. Data represent the mean ± SEM of five experiments performed in triplicates.

One potential consequence of receptor–receptor interactions is an alteration in ligand affinity. To assess whether coexpression of the N1 and N2 subunits of the NMDAR affects the affinity of CB_1_R for its ligands, we performed a nonradioactive Homogeneous Time-Resolved FRET (HTRF) assay using CELT-335, a potent and selective fluorescent CB_1_R ligand.^35^ SNAP-tag technology (see Materials and Methods) was applied in HEK-293T cells expressing the SNAP protein fused to the CB_1_R. Under these conditions, the *K*_*i*_ value of ACEA, a selective CB_1_R agonist competing with CELT-335 for binding, was 4.8 nM—consistent with previously reported values (Figure 1C). However, when the same assay was conducted in cells coexpressing SNAP-CB_1_R along with the N1 and N2 subunits of the NMDAR, the *K*_*i*_ value for ACEA increased to 17.7 nM (Figure 1D). This reduced affinity suggests that the presence of NMDAR subunits induces conformational changes in CB_1_R, possibly due to direct receptor–receptor interaction. As demonstrated below, the coexpression of N1 and N2 subunits leads to functional outputs that support the notion that conformational changes in CB_1_Rs result from its interaction with fully assembled NMDA receptors.

### Further Evidence of Receptor–Receptor Interactions by Assessing Signaling in HEK-293T Cells

Given that CB_1_R is a *G_i_*-coupled GPCR and its activation leads to inhibit adenylyl cyclase activity and reduce intracellular cAMP levels, we first assessed the cAMP signaling pathway. In HEK-293T cells coexpressing CB_1_R and NMDAR N1 and N2 subunits, stimulation with the CB_1_R-selective agonist ACEA led to a ∼40% decrease in forskolin-induced cAMP levels (Figure 2A), consistent with canonical CB_1_R signaling. As expected, NMDA alone had no effect on cAMP, since NMDAR does not engage this pathway. Interestingly, costimulation with ACEA and NMDA did not alter the magnitude of the cAMP response compared to ACEA alone. However, the CB_1_R-mediated effect was blocked not only by the CB_1_R antagonist SR141617 (rimonabant) but also by MK-801, a noncompetitive NMDAR antagonist. This effect, known as cross-antagonism, suggests functional coupling between the two receptors and is considered a key signature of receptor–receptor interactions.^36–39^

**Figure 2:**
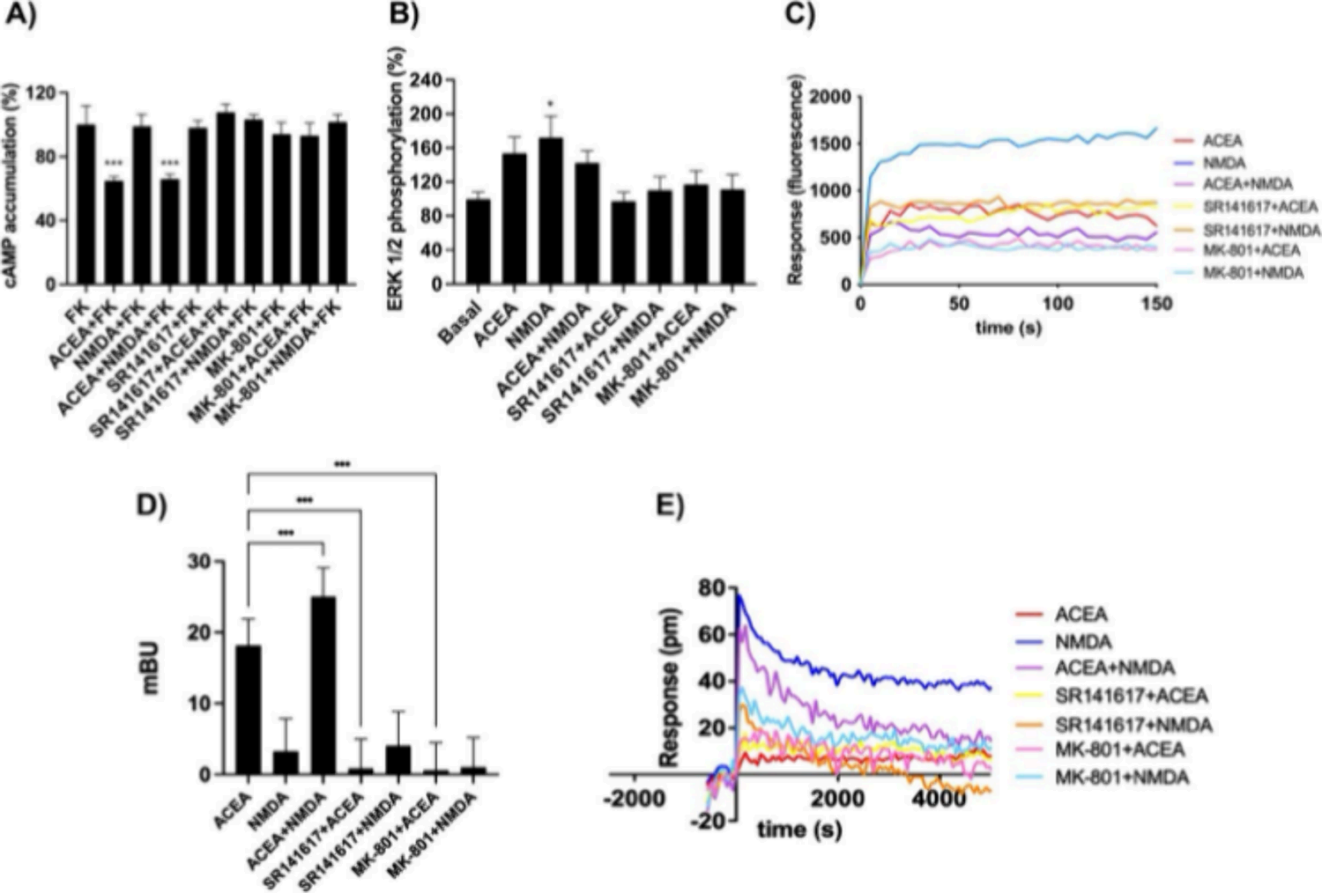
Functional characterization of CB_1_ and NMDA receptors in transfected HEK-293T cells. (A, B, D) HEK-293T cells were cotransfected with cDNAs encoding CB_1_R (0.3 μg), N1 (0.5 μg), and N2B (0.5 μg). Cells were pretreated with selective antagonists (1 μM SR141716—rimonabant—for CB_1_R or 1 μM MK-801 for NMDAR), followed by stimulation with agonists (100 nM ACEA and/or 15 μM NMDA). Functional responses were assessed by measuring cAMP levels (A), ERK1/2 phosphorylation (B), and dynamic mass redistribution (DMR) (E). (D) β-Arrestin-2 recruitment was measured in cells expressing β-arrestin-2-Rluc, CB_1_R-YFP, N1, and N2B, following the same treatment protocol as in (B). Increase in cytosolic calcium was measured in HEK-293T cells transfected with cDNA coding CB_1_R (0.3 μg), N1 (0.5 μg), N2B (0.5 μg), and the 6GCamMP calcium sensor (0.5 μg), under identical antagonist/agonist treatment conditions. Data are presented as mean ± SEM from six to eight independent experiments performed in triplicates. One-way ANOVA followed by Bonferroni’s post hoc test was used for statistical analysis (*p < 0.05, ***p < 0.001 vs 500 nM forskolin (FK) in cAMP assays, vs basal in pERK1/2 assays, or vs ACEA treatment in β-arrestin-2 recruitment assays).

To further assess functional crosstalk, we focused on the MAPK signaling pathway that may be engaged upon agonist-induced activation of the CB_1_R and, also, of the NMDAR. Although both CB_1_R and NMDAR stimulation induced ERK1/2 phosphorylation (Figure 2B), coactivation did not lead to an additive response. ERK1/2 phosphorylation induced by NMDA was inhibited not only by MK-801, but also by rimonabant, and vice versa. Therefore, cross-antagonism was observed across both signaling pathways analyzed.

Cytosolic calcium levels signaling induced by NMDA were determined. In HEK-293T cells coexpressing CB_1_R and NMDAR N1 and N2 subunits, NMDAR reconstitution occurred and its stimulation produced a robust cytosolic calcium increase. ACEA partially attenuated the NMDA-induced calcium response (Figure 2C), indicating a *negative crosstalk*. Cross-antagonism was again observed in this assay, reinforcing the idea of molecular and functional links between both receptors.

To explore whether cross-modulation extends to CB_1_R receptor desensitization and internalization, we evaluated β-arrestin-2 recruitment upon receptor activation by ACEA. Using a BRET-based β-arrestin-2 recruitment assay, we found that the agonist of the NMDAR significantly enhanced ACEA-induced β-arrestin-2 binding to CB_1_R (Figure 2D), suggesting that NMDAR activity may contribute to shortening the duration of the effect of cannabinoids acting on CB_1_R. Again, a cross-antagonism was detected.

Finally, dynamic mass redistribution (DMR) assays were performed to monitor global cellular responses such as receptor activation and cytoskeletal reorganization. The DMR data mirrored the results of the other signaling assays studied, displaying clear patterns of negative crosstalk and cross-antagonism between CB_1_R and NMDAR (Figure 2E).

Altogether, these findings provide strong functional evidence of receptor cross-modulation between CB_1_R and NMDAR, reinforcing the hypothesis that these receptors form functional complexes with the capacity to modulate each other’s signaling.

### Evidence of CB_1_–NMDA Receptor–Receptor Interactions in Microglia

To investigate the expression of CB_1_R-N1 subunit complexes in primary microglia from mouse brain, a proximity ligation assay (PLA) was performed. Using this technique, interactions of the two proteins were observed as punctate red clusters (Figure 3A,B). The staining was observed in a high percentage of naïve/resting microglial cells (>80%), with an average of seven puncta per positive cell. Furthermore, microglia activated using 1 μM LPS and 200 U/mL IFN-γ showed an important increase in the CB_1_R-N1 complex expression, with >90% of cells showing red clusters and an average of 18 puncta per positive cell. Primary microglia (naïve/resting) obtained from the brain of the APP_Sw/Ind_ transgenic mouse model of AD also showed a high percentage of cells (>80%) expressing clusters with an average of 10 puncta per positive cell (Figure 3A,B).

**Figure 3:**
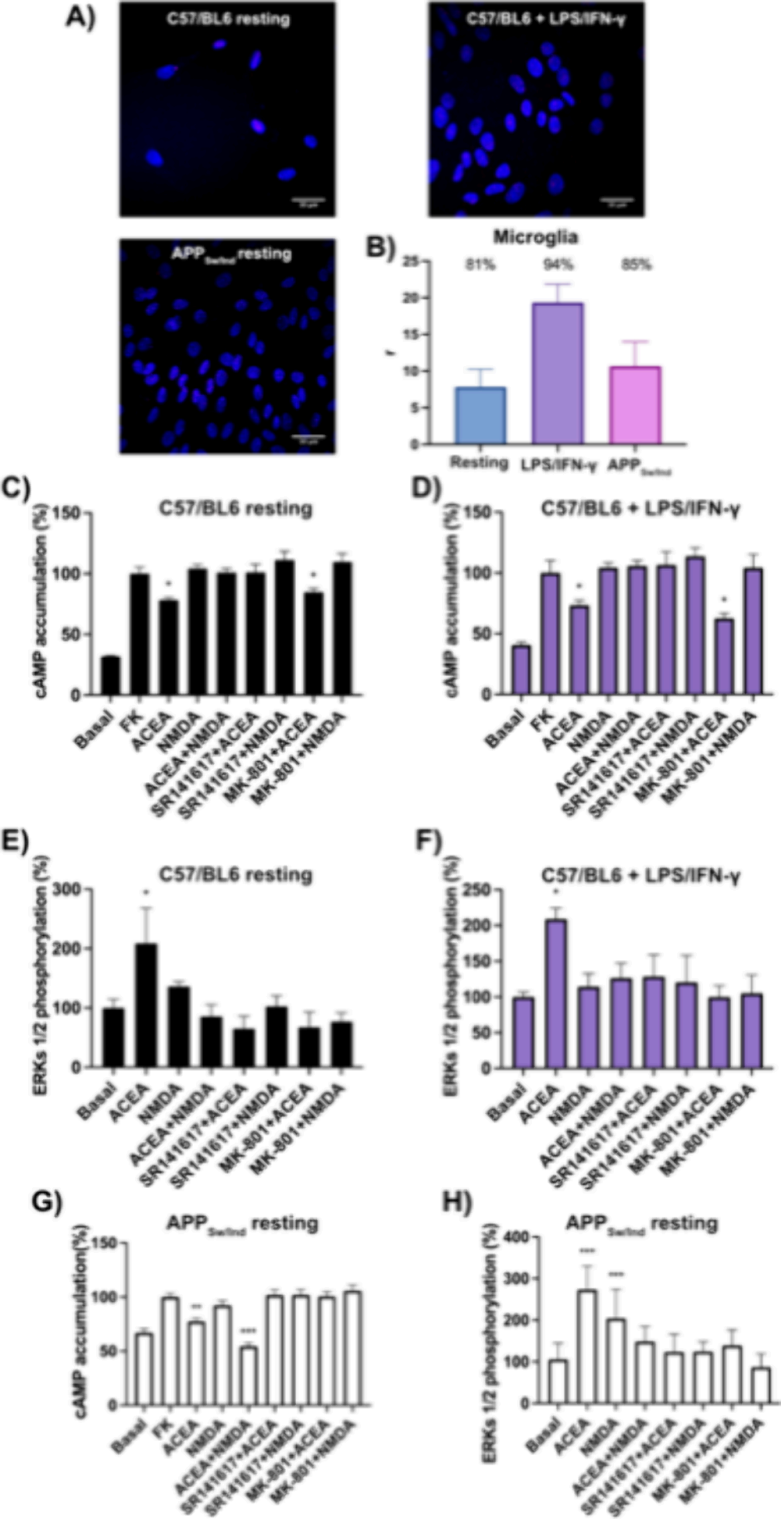
Detection of CB_1_R-N1 subunit complexes and assessment of receptor functional crosstalk in primary microglia. (A) Proximity ligation assay (PLA) was performed in primary microglia from C57BL/6 mice under naïve/resting conditions and after 1 μM LPS and 200 U/mL IFN-γ, and in naïve/resting microglia from APP_Sw/Ind_ mice. CB_1_R-N1 complexes appear as red puncta in confocal microscopy images (merged sections shown). Nuclei were stained with Hoechst (blue). Scale bar = 30 μm. (B) Quantification of PLA signals: number of red puncta per cell (*r*) and percentage of cells exhibiting puncta (values shown above bars). Data represent mean ± SEM (*n* = 6). Statistical analysis was performed using one-way ANOVA followed by Bonferroni’s post hoc test (**p < 0.01 vs resting/naïve microglia). (C–H) Functional characterization. Cells from nontrangenic mice, naïve/resting (C, E) or activated with LPS + IFN-γ (D, F), and from APP_Sw/Ind_ mice (naïve/resting) were analyzed in parallel (G, H). Cells were pretreated with selective antagonists (1 μM SR141716A or 1 μM MK-801) followed by agonist stimulation (100 nM ACEA and/or 15 μM NMDA). (C, D, G) cAMP levels in cells pretreated with forskolin (FK); (E, F, H) ERK1/2 phosphorylation. Data are expressed as mean ± SEM from 6–12 independent experiments. Statistical analysis was performed using one-way ANOVA followed by Bonferroni’s post hoc test (*p < 0.05, **p < 0.01, ***p < 0.001 vs forskolin -FK-in cAMP assays or vs basal in ERK1/2 phosphorylation assays).

The presence of complexes of CB_1_R and the N1 subunit of the NMDAR in microglia prompted us to investigate the effect of activating the CB_1_ and/or the NMDA receptors. First, we measured intracellular cAMP levels in both naïve (resting) and activated (under inflammatory stimuli) microglia from nontransgenic animals. Treatment with the CB_1_R agonist ACEA significantly attenuated forskolin-induced cAMP elevation, consistent with canonical *G_i_*-mediated signaling. However, this effect was counteracted by NMDA, which prevented the CB_1_R-mediated reduction in cAMP levels (Figure 3C,D). Notably, the cross-antagonism observed in HEK-293T cells was neither found in resting nor activated microglia from nontransgenic animals. In contrast, naïve (resting) microglia derived from the APP_Sw/Ind_ mouse model of AD exhibited a fairly distinct pattern because NMDA not longer blocked *G_i_*-mediated signaling upon CB_1_R activation by ACEA (Figure 3G). These differences may reflect variations in heteromer composition or the involvement of additional proteins in the receptor complex under overexpression of the mutant form of human amyloid precursor protein.

Next, we examined the mitogen-activated protein kinase (MAPK) signaling pathway by measuring ERK1/2 phosphorylation. In both naïve (resting) and activated microglia from nontransgenic mice, NMDA blocked CB_1_R-induced ERK1/2 phosphorylation. In addition cross-antagonism was found as CB_1_R-mediated effects were inhibited not only by the selective CB_1_R antagonist rimonabant but also by the NMDAR antagonist MK-801 (Figure 3E,F). In primary naïve microglia from APP_Sw/Ind_ mice, both ACEA and NMDA led to a significant increase in ERK1/2 phosphorylation. However, cotreatment did not significantly increase ERK1/2 phosphorylation over basal levels. This result and the reciprocal cross-antagonism (Figure 3H) are evidence of cross-modulation likely derived from a direct interaction between the two receptors.

### Functional CB_1_-NMDA Receptor–Receptor Crosstalk in Primary Cortical and Hippocampal Neurons from Nontransgenic Mice

To investigate the presence of CB_1_R-N1 subunit complexes in primary neurons from the cortex and hippocampus, regions particularly vulnerable in AD, PLA was undertaken. Red fluorescent clusters indicative of protein complexes were detected (Figure 4A,B). In cortical neurons, more than 20% of cells exhibited red clusters, with an average of ∼2.5 puncta per positive cell. In hippocampal neurons, >30% of cells displayed red puncta, also averaging ∼2.5 puncta per positive cell. These findings align with a higher CB_1_R expression level in hippocampal compared to cortical neurons. Notably, pretreatment with Aβ_1–42_ oligomers (500 nM, 48 h) significantly increased both the proportion of neurons displaying CB_1_R-N1 complexes and the number of puncta per positive cell. Following Aβ_1–42_ exposure, >40% of cortical neurons and >60% of hippocampal neurons displayed red clusters, with an average of ∼4 puncta per positive cell in both regions. Negative controls lacking the anti-CB_1_R primary antibody exhibited minimal signal (<10% of cells with red puncta), confirming the specificity of the staining. These results demonstrate the presence of CB_1_R-N1 complexes in both cortical and hippocampal neurons, with a marked upregulation following Aβ_1–42_ treatment.

**Figure 4:**
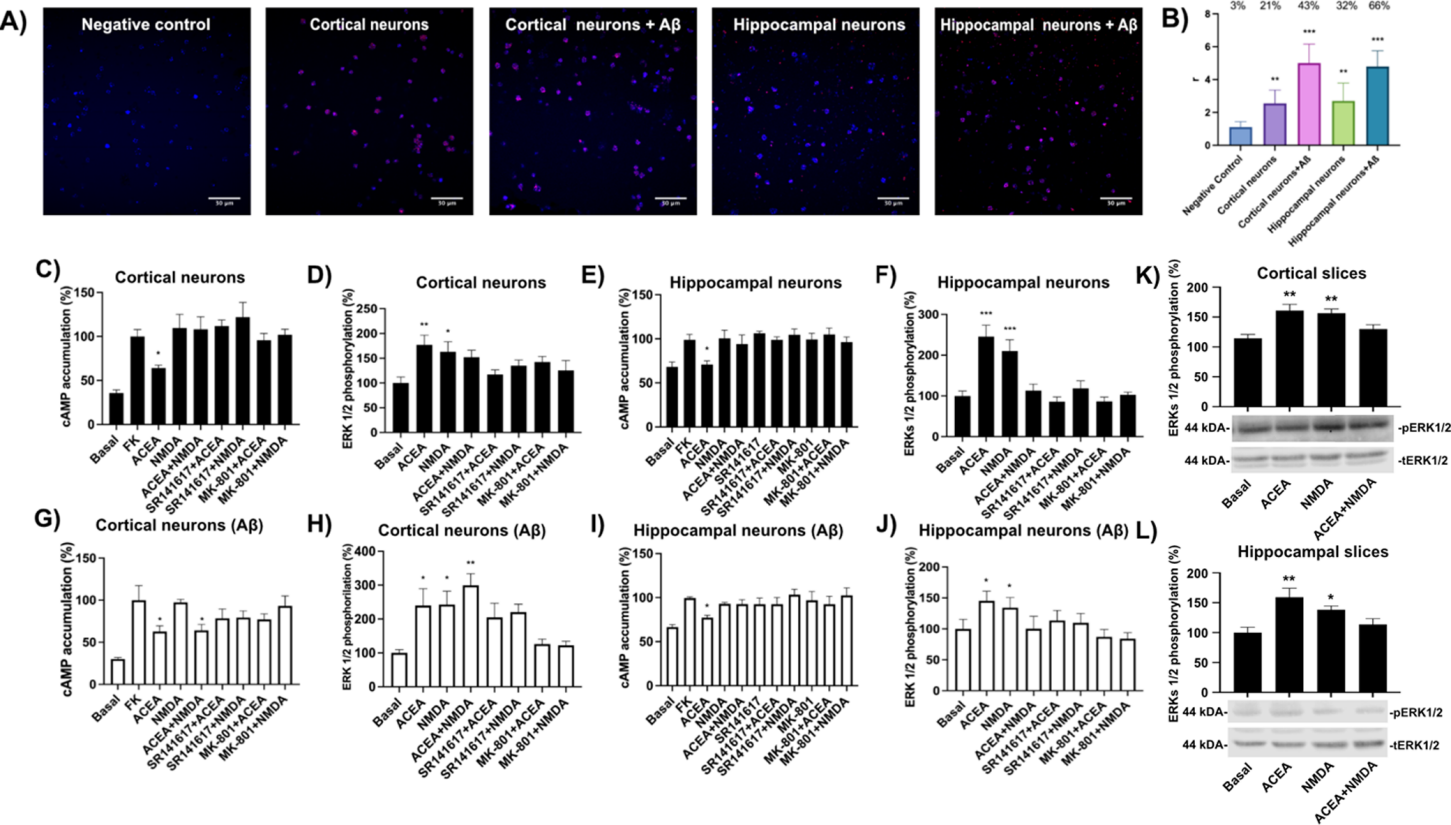
Detection of CB_1_R-N1 subunit complexes and assessment of functional crosstalk and neuroprotection against Aß_1–42_ in primary neurons. (A) Proximity ligation assay (PLA) was performed in primary cortical and hippocampal neurons from C57BL/6 mice treated or not with Aβ1–42 oligomers (500 nM, 48 h). A negative control condition omitting primary antibodies was included. Nuclei were stained with Hoechst (blue), and CB_1_R–NMDAR complexes appear as red puncta in confocal microscopy images (merged sections shown). Scale bar = 30 μm. (B) Quantification of PLA signal: number of red puncta per cell (*r*) and percentage of cells exhibiting puncta (indicated above each bar). Data are shown as mean ± SEM (*n* = 6). One-way ANOVA followed by Bonferroni’s post hoc test was used for statistical analysis (**p < 0.01, ***p < 0.001 vs negative control). (C–J) Functional interaction between CB_1_R and NMDAR was evaluated in primary cortical and hippocampal neurons from untreated (C–F) or Aβ_1–42_-treated (G–J) C57BL/6 mice. Cells were pretreated with selective antagonists (1 μM SR141716A (rimonabant) for CB_1_R or 1 μM MK-801 for NMDAR), followed by agonist stimulation (100 nM ACEA and/or 15 μM NMDA for NMDAR). Determination of cAMP levels is shown in (C, E, G) and (I). Determination of ERK1/2 phosphorylation is shown in (D, F, H, J). Data represent mean ± SEM from 8–12 independent experiments. One-way ANOVA followed by Bonferroni’s post hoc test was applied for statistical analysis (*p < 0.05, **p < 0.01, ***p < 0.001 vs forskolin (FK) in the cAMP assay or vs basal in the ERK1/2 phosphorylation assay). (K–L) ERK1/2 phosphorylation was determined in hippocampal and cortical slices of C57BL/6 mice treated with selective agonists. Data represent the mean ± SEM from six independent experiments. One-way ANOVA followed by Bonferroni’s post hoc test was applied for statistical analysis (*p < 0.05, **p < 0.01, vs basal in the ERK1/2 phosphorylation assay).

To investigate the functional consequences of CB_1_R–NMDAR interactions, we first assessed intracellular cAMP levels. In both cortical and hippocampal neurons, stimulation with the CB_1_R agonist ACEA reduced forskolin-induced cAMP accumulation, consistent with *G_i_* protein coupling. This effect was abolished by coapplication of NMDA, suggesting a negative crosstalk between the receptors (Figure 4C,E). Activation of either receptor alone induced ERK1/2 phosphorylation; however, simultaneous stimulation with both agonists failed to produce additive or synergistic effects. In hippocampal neurons, cotreatment with ACEA and NMDA did not enhance ERK1/2 phosphorylation beyond basal levels (Figure 4D,F). Cross-antagonism, a hallmark of receptor–receptor interactions, was evident in both signaling pathways in cortical and hippocampal neurons. Antagonism of either receptor disrupted the response mediated by the other, supporting functional interplay between the CB_1_R and NMDAR systems.

Hippocampal neurons treated with Aβ_1‑42_ exhibited signaling profiles similar to those of untreated cells, with cross-antagonistic interactions remaining intact (Figure 4I,J). Cross-antagonism was consistently observed in all tested neurons, regardless of cortical or hippocampal origin, Aβ_1‑42_ exposure, or assay type (cAMP or ERK1/2 phosphorylation; Figure 4C–J). Furthermore, PLA signals indicative of CB_1_R–N1 subunit interactions were significantly enhanced following Aβ_1‑42_ treatment in both cortical and hippocampal neurons, suggesting that the amyloid peptide promotes heteromer formation (Figure 4A,B). However, in cortical neurons exposed to Aβ_1‑42_, NMDA no longer inhibited the CB_1_R-mediated suppression of forskolin-induced cAMP (Figure 4G). This dissociation between receptor complex expression and functional output implies the involvement of additional molecular mechanisms modulating CB_1_R–NMDAR signaling post-Aβ_1‑42_ exposure. Notably, in Aβ_1‑42_-treated cortical neurons, the combined effect of ACEA and NMDA on signaling responses diverged from that observed with ACEA and MK-801, unlike in untreated cells where both treatments yielded similar outcomes (Figure 4G,H). Importantly, experiments performed in cortical and hippocampal slices revealed a negative crosstalk, supporting the presence of CB_1_R–NMDAR heteromers that are functionally interacting in the native brain tissue (Figure 4K,L).

### ACEA Reduces NMDA-Mediated Neurotoxicity

To assess whether activation of CB_1_R mitigates neurotoxicity induced by NMDA, neurite formation was evaluated in primary cortical and hippocampal neurons treated with ACEA, NMDA, or a combination of both. Immunocytochemistry was performed using an anti-Nectin antibody to visualize neurite patterning, and an anti-F-actin antibody conjugated to Alexa Fluor 488 to label neurons. The results showed that NMDA treatment significantly reduced neurite formation, while ACEA alone had no detectable effect compared to vehicle-treated controls in both cortical and hippocampal neurons. Interestingly, cotreatment with NMDA and ACEA partially counteracted the neurite loss induced by NMDA, indicating a neuroprotective effect of CB_1_R activation (Figure 5). To further explore this protective role, the same experimental paradigm was applied to neurons pretreated with Aβ_1–42_ (500 nM) for 48 h. Aβ_1–42_ exposure led to a marked reduction in neurite formation, which was further exacerbated by NMDA treatment. Notably, ACEA treatment fully prevented the neurite loss induced by Aβ_1–42_ and partially reversed the additional effect caused by NMDA (Figure 5). Together, these findings suggest that CB_1_R activation can protect cortical and hippocampal neurons from neurotoxic insults induced by NMDA and Aβ_1–42_, likely through mechanisms related to the preservation of neuronal plasticity.

**Figure 5:**
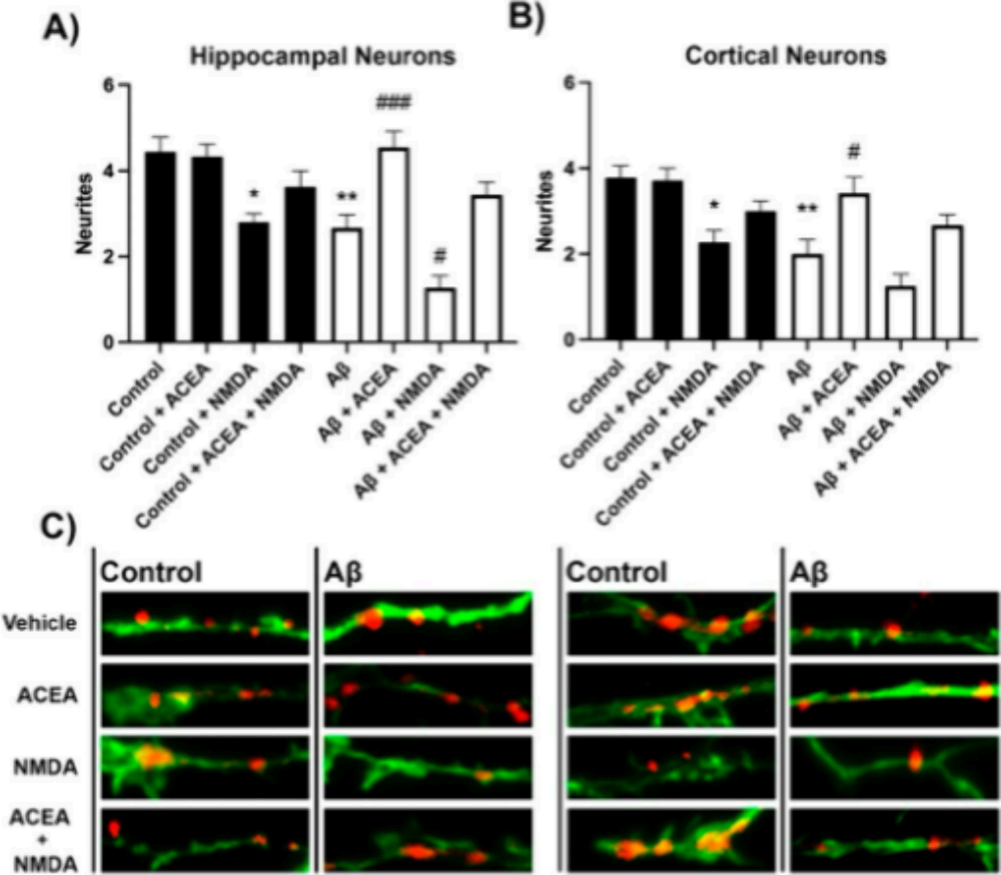
Effect of ACEA and NMDA on neurite patterning in primary cortical and hippocampal neurons treated with Aß_1–42_. On DIV 10, cortical and hippocampal primary cultures of neurons were treated with Aß 1–42 (500 nM) for 48 h. On DIV 11, neurons were stimulated with vehicle, ACEA (100 nM), NMDA (15 μM) or both. Neurite patterning was detected by immunocytochemistry using an anti-Nectin antibody (Abcam, 1/1000). Neurons were detected with the 3 anti-F-actin antibody fused to an Alexa 488 fluorophore (ThermoFisher, 1/400). **A-B.** Quantification was performed over dendritic segments of 15 μm. **C.** Representative images for each condition. Red represents neurite formation. Values are the mean ± SEM of 12 independent experiments performed in duplicates. One-way ANOVA followed by Bonferroni’s multiple comparison post hoc test were used for statistical analysis *p < 0.05, **p < 0.005, ***p < 0.001 versus vehicle treatment.

### Functional CB_1_-NMDA Receptor–Receptor Crosstalk in Primary Neurons from an APP_Sw/Ind_ Mouse Model

After showing evidence that treatment with Aβ_1–42_ enhances the expression of CB_1_R-N1 complexes in primary neurons of nontransgenic mice, PLA allowed to identify a higher amount of CB_1_R-N1 complexes in neurons from the APP_Sw/Ind_ mouse AD model. The percentages of cells expressing these protein complexes were 56 and 73 in cortical and hippocampal neurons, respectively, with a similar number of puncta per positive neuron (ca. 4.5 puncta). Such expression is even higher than that displayed by neurons from nontransgenic mice after the treatment with Aß_1–42_. cAMP level determination assays were conducted in primary cortical and primary hippocampal neurons of the APP_Sw/Ind_ mouse. When analyzing the CB_1_R–NMDAR functionality in cortical and in hippocampal neurons from APP_Sw/Ind_ mice, it was observed that NMDA minimally affected the ACEA-induced decrease of cAMP while cross-antagonism was maintained (Figure 6A,B). The cross-antagonism was also detected in the ERK1/2 phosphorylation assays (Figure 6C,D). Overall, the results were similar to those obtained in primary neurons of nontransgenic mice treated with Aß_1–42_.

**Figure 6:**
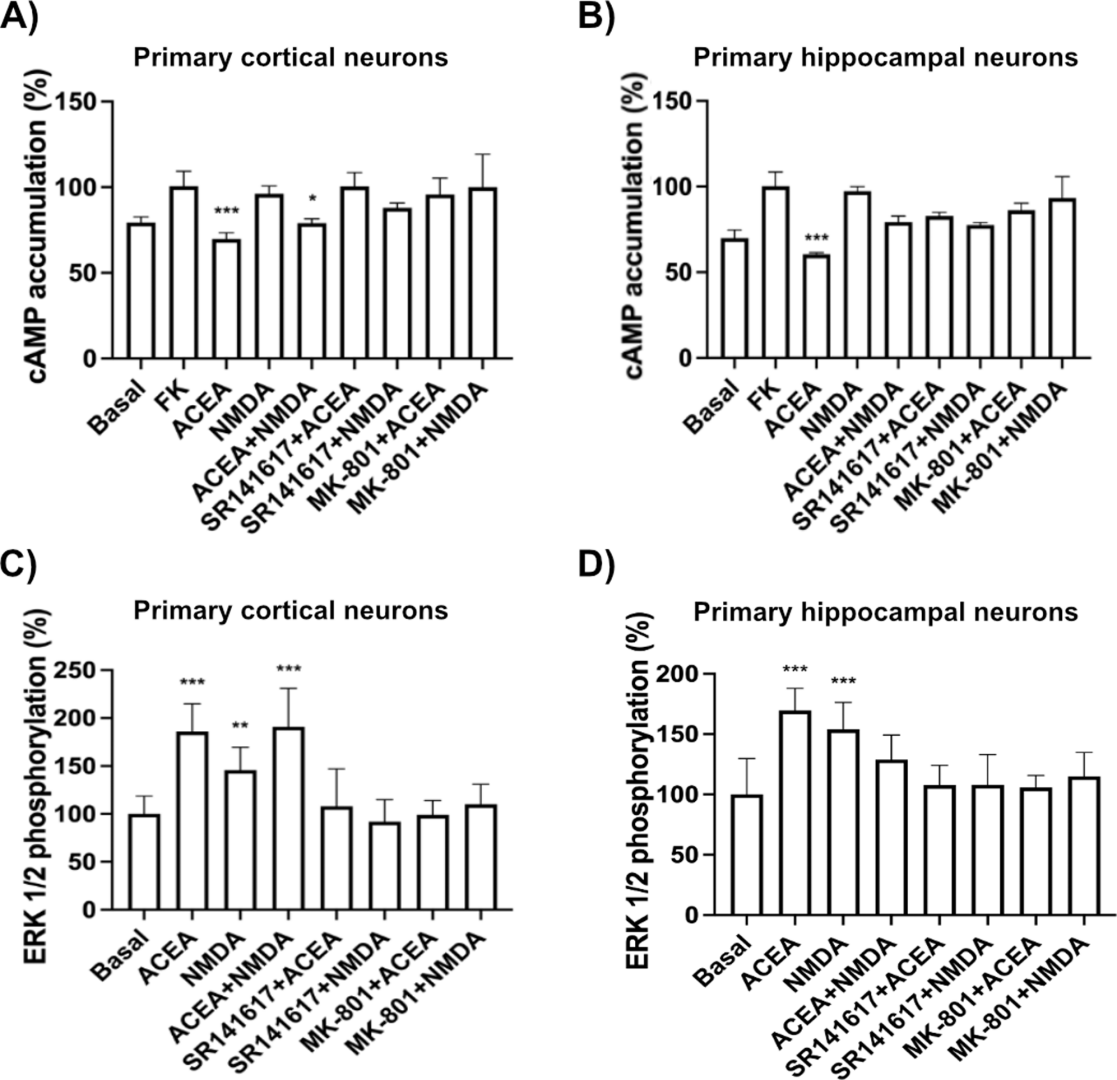
Assessment of functional crosstalk and neuroprotection against Aß_1–42_ in primary cortical and hippocampal neurons from APP_Sw/Ind_ mice. (A–D) Functional analysis in primary cortical and hippocampal neurons from APPSw/Ind mice pretreated with selective antagonists (1 μM SR141716A (rimonabant) for CB_1_R or 1 μM MK-801 for NMDAR), followed by agonist stimulation (100 nM ACEA and/or 15 μM NMDA). (A, B) cAMP levels were determined in cells previously treated with 500 nM forskolin (FK). (C, D) Erk1/2 phosphorylation. Results are shown as mean ± SEM from eight independent experiments. One-way ANOVA followed by Bonferroni’s post hoc test was used for statistical analysis (*p < 0.05, **p < 0.01, ***p < 0.001 vs forskolin-treated condition in cAMP assays or vs basal in ERK1/2 phosphorylation assays).

## Discussion

AD is the most common form of dementia, accounting for over 60% of cases worldwide. Despite significant research efforts, its etiology remains unclear, and current treatments are only symptomatic. One such treatment is memantine, an NMDAR antagonist, which underscores the relevance of NMDA receptors. NMDARs exert protective effects when synaptically localized but can be neurotoxic when activated at extrasynaptic sites.^8,40,41^

Sánchez-Blázquez et al. (2013) proposed that CB_1_Rs counteract NMDA-mediated excitotoxicity via direct interaction with the N1 subunit of NMDAR, stabilized by histidine triad nucleotide-binding protein 1 (HINT1).^24^ This interaction allows CB_1_Rs to reduce surface NMDARs through cointernalization, diminishing excitotoxic signaling (e.g., calcium influx, NO production, and zinc mobilization). The physiological function depends on the structural integrity of the complex, which is disrupted by PKA activation or in HINT1-deficient mice. These findings established CB_1_R as a context-sensitive modulator of glutamatergic toxicity.^42,43^ These previous studies focused primarily on neurons and nitric oxide, overlooking microglia, the main producers of NO in the CNS and key players in neuroinflammation and AD. Here, we present the first evidence of CB_1_R–NMDAR interactions in primary microglia, including in naïve/resting and LPS/IFN-γ-activated states. PLA revealed CB_1_R-N1 complexes in microglia from nontransgenic mice, with increased expression under inflammatory stimuli. Functional assays showed a bidirectional crosstalk in cAMP and MAPK pathways, highlighting that these interactions are not neuron-specific and are influenced by cellular context.

Previous studies focused primarily on neurons and nitric oxide, overlooking microglia, the main producers of NO in the CNS and key players in neuroinflammation and AD. Here, we present the first evidence of CB_1_R–NMDAR interactions in primary microglia, including in resting and LPS/IFN-γ-activated states. PLA revealed an increased expression of CB_1_R–N1 complexes under inflammatory stimuli. Functional assays showed a bidirectional crosstalk in cAMP and MAPK pathways, highlighting that these interactions are not neuron-specific and are influenced by cellular context.

Interestingly, while cross-antagonism between CB_1_R and NMDAR was consistently observed in HEK-293T cells and primary neurons, it was notably absent in naïve microglia from nontransgenic mice, yet clearly present in microglia derived from APP_Sw/Ind_ mice. This finding suggests that CB_1_R–NMDAR functional interactions are differentially regulated in AD, potentially reflecting disease-driven alterations in microglial activation states. In a previous study, we reported that CB_1_R expression is upregulated in naïve microglia from APP_Sw/Ind_ mice, which exhibited a functional profile resembling that of activated microglia from wild-type animals.^26^ These observations support the idea that, in the AD context, microglia may adopt a chronically activated phenotype, likely aligned with an M2-like, neuroprotective state.

We further confirmed the presence of CB_1_R–N1 complexes in cortical and hippocampal neurons, with Aβ_1–42_ treatment enhancing their formation. Despite increased expression, functional crosstalk varied with region, treatment, and genotype, indicating that receptor abundance alone does not determine interaction strength. In cortical neurons, NMDA failed to block ACEA-induced cAMP inhibition after Aβ_1–42_ exposure, whereas hippocampal neurons retained classical negative crosstalk patterns.

Importantly, the functional outcomes of CB_1_R–NMDAR interactions are strongly context-dependent, suggesting the involvement of additional proteins within the signalosome that modulate these interactions. This is exemplified by two key observations. First, cross-antagonism, a hallmark of receptor–receptor interaction, was consistently observed across conditions, with one notable exception: naïve microglia from nontransgenic mice, where it was absent. Second, the negative modulation of CB_1_R *G*_*i*_ coupling by NMDA varied by cell type and disease context. This inhibitory effect was detected in hippocampal neurons, regardless of Aβ exposure or transgenic status, but was not observed in cortical neurons under any condition. In microglia, NMDA-mediated disruption of CB_1_R *G*_*i*_ coupling was evident in both resting and activated cells from nontransgenic mice, but this effect was lost in resting microglia from the APP_Sw/Ind_ model. These cell-type-specific differences highlight the complexity and plasticity of the CB_1_R–NMDAR signalosome and point to a dynamic interplay modulated by yet-to-be-identified scaffold or regulatory proteins.

These findings suggest that signaling via the CB_1_R–NMDAR is shaped by multiple factors: cell type, developmental stage, disease context, and the molecular composition of the signalosome.^44,45^ For example, changes in NMDAR subunit composition (e.g., N2 vs N3) or availability of scaffold proteins and kinases (e.g., β-arrestins, CaMKII, and PKA) could modulate receptor conformation and signaling outcomes.^46–48^ Supporting this, binding assays showed reduced ACEA affinity when CB_1_R was coexpressed with N1 and N2 subunits, suggesting conformational shifts.

Notably, CB_1_R activation rescued neurite loss induced by NMDA and Aβ_1–42_, highlighting its protective role. This aligns with the hypothesis that CB1R–NMDAR heteromers act as context-dependent signalosomes capable of integrating extracellular cues with intracellular responses.

Overall, our findings reinforce the therapeutic potential of targeting CB_1_R–NMDAR interactions in AD and neuroinflammation.^49–51^ Unlike direct NMDAR antagonists, which can disrupt normal synaptic activity,^52,53^ CB_1_R agonists offer a more selective approach by modulating pathological signaling via receptor–receptor interactions. Future therapies might aim to stabilize or bias these complexes using allosteric modulators tailored to preserve physiological glutamatergic function while attenuating excitotoxicity.

## Conclusions

This study demonstrates that CB_1_ and NMDA receptors form functional heteromeric complexes in both neurons and microglia, under physiological conditions and in pathological contexts such as AD models. These interactions are not merely structural but lead to bidirectional signaling crosstalk, notably including cross-antagonism across key intracellular pathways. CB_1_R–NMDAR complexes do not consistently predict their functional impact, pointing to the critical influence of the surrounding signalosome composition. This suggests that additional, cell-specific molecular components govern the signaling outcomes of these receptor complexes.

Our results show that functional complexes involving CB_1_ and NMDA receptors are highly plastic and context-dependent, integrating diverse intracellular components that shape their signaling outputs. This complexity explains the cell- and condition-specific effects of cannabinoid signaling and highlights the therapeutic potential of selectively targeting CB_1_R within such receptor complexes. The ability of CB_1_R to modulate NMDAR activity indirectly, via receptor–receptor interactions, provides a compelling therapeutic avenue. Targeting CB_1_R within these defined signalosomes, rather than using global NMDAR antagonists, may allow for selective attenuation of excitotoxicity, preservation of neuronal structure, and modulation of neuroinflammatory processes in AD and related neurodegenerative disorders.

## Data Availability

Data can be obtained from the corresponding author, G.N., upon reasonable request.

## Abbreviations

A_2A_R: adenosine A_2A_ receptor
Aβ: ß-amyloid
AD: Alzheimer’s disease
APP: amyloid precursor protein
AMPA: α-amino-3-hydroxy-5-methyl-4-isoxazole propionic acid
BRET: Bioluminescence Energy Transfer
CB_1_R: cannabinoid receptor type 1
CB_2_R: cannabinoid receptor type 2
D_1_R: dopamine D_1_ receptor
DMEM: Dulbecco’s modified Eagle’s medium
ERK1/2: extracellular signal-regulated kinase 1/2
FK: forskolin
GPCR: G protein-coupled receptor
H_3_R: histamine H_3_ receptor
HINT1: histidine triad nucleotide-binding protein 1
HTRF: homogeneous time-resolved FRET
IFN-γ: interferon-γ
LPS: lipopolysaccharide
MAPK: mitogen-ativated protein kinase
NMDA: *N*-methyl-d-aspartate
NMDAR: NMDA receptor
Rluc: Renilla luciferase
YFP: yellow fluorescent protein
